# Diagnostic Colonoscopy Leading to Perforated Appendicitis: A Case Report and Systematic Literature Review

**DOI:** 10.1155/2016/1378046

**Published:** 2016-11-17

**Authors:** Daniel Paramythiotis, Konstantinia Kofina, Vasileios Papadopoulos, Antonios Michalopoulos

**Affiliations:** First Propedeutic Surgical Department, Aristotle University of Thessaloniki, Thessaloniki, Greece

## Abstract

*Introduction*. Intestinal perforation is a known complication after colonoscopy. However, appendiceal involvement with inflammation and perforation is extremely rare and only 37 cases of postcolonoscopy appendicitis have been reported so far. We describe a case of perforated appendicitis 24 hours after colonoscopy that was treated successfully in our Department.* Case Report*. A 60-year-old female patient underwent a colonoscopy during the investigation of nontypical abdominal pain without pathologic findings. 24 hours after the examination she presented gradually increased right lower quadrant abdominal pain and a CT scan was performed, showing an inflammation of the appendiceal area with free peritoneal air. Through laparotomy, perforated appendicitis was diagnosed and an appendectomy was performed. The patient was discharged on the tenth postoperative day in good health condition.* Discussion*. The characteristics of all cases reported in the literature are described, including our case. Perforated appendicitis soon after a colonoscopy is a rare, but serious complication; therefore, it is crucial to be included in the differential diagnosis of postcolonoscopy acute abdominal pain.

## 1. Introduction

Colonoscopy is a common procedure used by the gastroenterologists in order to investigate abnormal conditions of the colon and the distal small intestine [1]. Serious complications are not considered as frequent, but they include a wide range of conditions, from pain, bleeding, and inflammation to perforation, cardiopulmonary complications, and even death [2].

Perforation is reported in 0.3% or lower of the cases examined. This complication could be caused by therapeutic interventions, air insufflation, mechanical forces, or movements of the scope [3]. The sigmoid is the most common location of perforation, possibly due to mechanical forces, diverticular disease, polyps, or the thinner colonic wall [4]. Women are considered more prone to perforation because of pelvic surgeries, diverticular disease, or increased colonic length.

Acute appendicitis has been referred to in the literature as a possible complication after a colonoscopy, probably in a higher rate than it was generally considered (until 2007 only 12 cases were reported) [5]. However, according to the ASGE guidelines of 2011, acute appendicitis is only referred to as a possible complication during a couple of cases, while rarer complications are described more thoroughly [6].

We present the case of a female patient that was submitted to diagnostic colonoscopy with normal results but presented with perforated appendicitis 24 hours after the examination. A thorough research of the literature and disclosure of the results has also been performed and recorded.

## 2. Case Report

**Figure 1:**
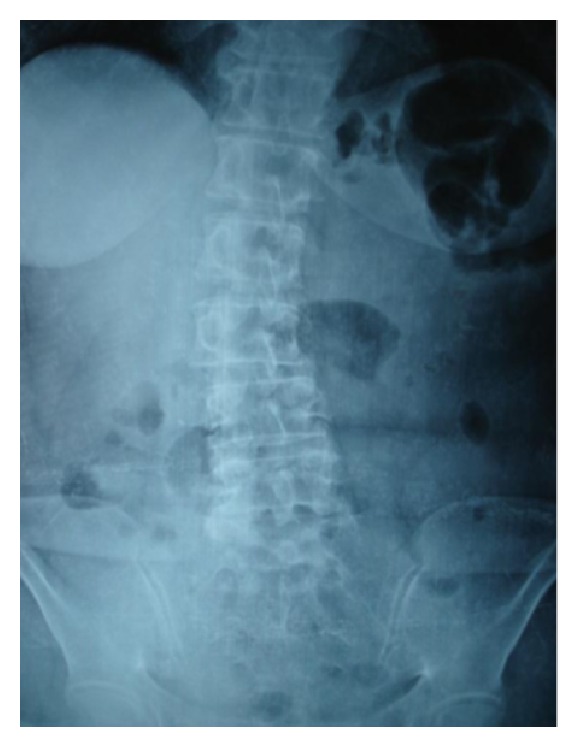
Abdominal X-ray on admission. Normal findings.

A 60-year-old woman was admitted to the Department of Internal Diseases after presenting mild pain at the lower abdomen and the right iliac fossa. The blood laboratory tests showed results within normal limits (WBC: 8.01 K/*μ*L, with 76.3% neutrophils) and imaging control through abdominal X-ray (Figure 1) and ultrasound did not reveal any abnormalities.

This initial condition of the patient could reassemble that of mild appendicitis; therefore examination by a surgeon was also realized. However, the patient did not present fever or anorexia, McBurney sign was negative within a couple of hours after the first examination, and tenderness in the lower abdomen had almost disappeared the next day, whereas the laboratory blood tests were still within normal limits.

**Figure 2:**
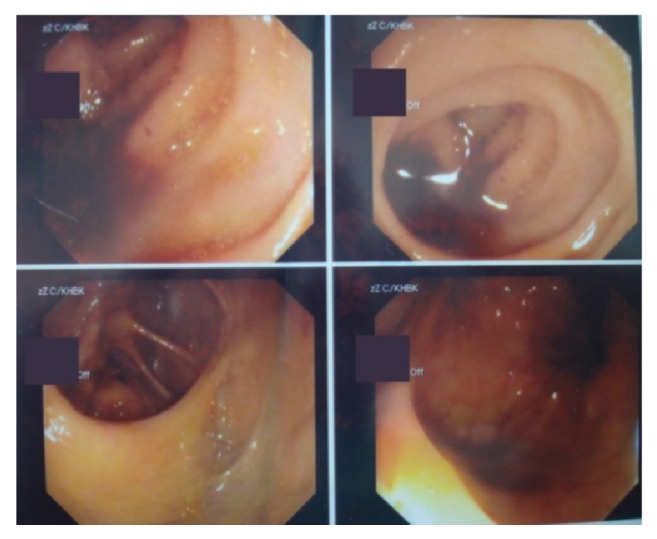
Imaging through colonoscopy. No signs of inflammation until the point of 10 cm proximally to the ileocecal valve.

Therefore, at first, the patient was treated by general practitioners, who considered her present condition as the symptoms of a possible bowel inflammation; this is the reason why they also considered necessary the initial treatment with rehydration and empirical antibiotic administration. Furthermore, the same clinical condition had occurred several times in the past and was treated conservatively with success. Based on this repeated clinical history, the colonoscopy was considered as the examination of choice for further investigation and was programmed immediately, in terms of the same admission. The procedure was uneventful and examination up to 10 cm proximally to the ileocecal valve did not lead to any pathologic findings (Figure 2).

**Figure 3:**
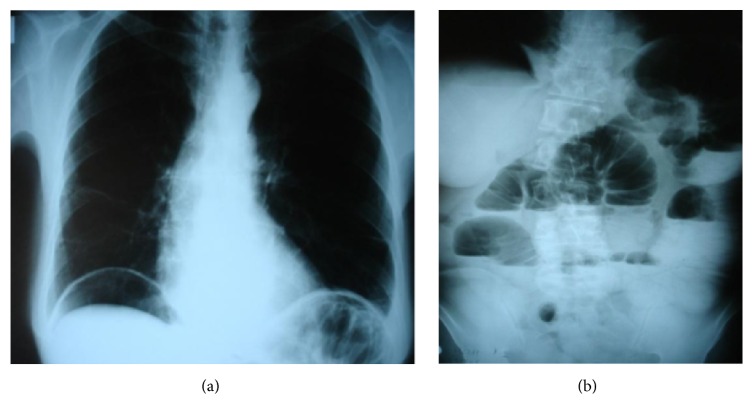
Abdominal X-ray after colonoscopy and clinical deterioration. Presence of free abdominal gas.

**Figure 4:**
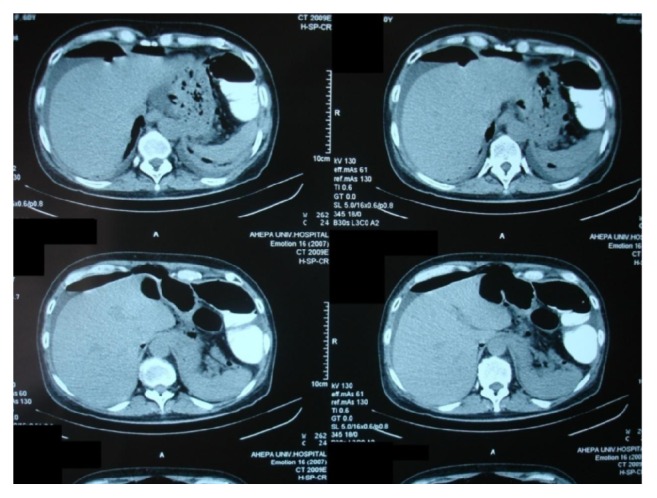
Abdominal CT scan. Perihepatic collection with free abdominal gas.

Mild abdominal pain reoccurred 10 hours after the colonoscopy, increased progressively, and presented as acute abdomen 24 hours after the procedure, especially to the right side, with flatulence, but still without fever or white blood cell count disturbance (WBC: 9.02 K/*μ*L, neutrophils: 78.6%). A new abdominal X-ray was performed, presenting free abdominal gas (Figures 3(a) and 3(b)). Surgical assessment was considered necessary, and imaging through computerized tomography was realized. The abdominal CT scan revealed diffuse inflammation not only in the area of the appendix and free gas in the peritoneal cavity, but also in the retroperitoneal space (Figure 4).

**Figure 5:**
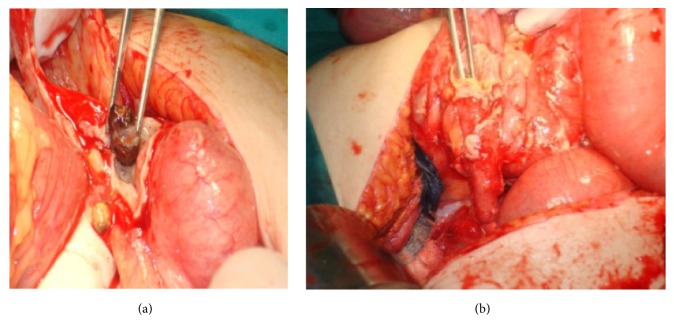
Intraoperative findings. Acute appendicitis with peritonitis. Presence of a fecalith.

Based on the condition and the imaging findings, an exploratory laparotomy was performed, during which perforated appendicitis with peritonitis was revealed (Figures 5(a) and 5(b)). An appendectomy was performed and the cavity was rinsed and drained thoroughly. The time interval between the presence of severe symptoms and surgery included the realization of X-ray and CT imaging and was about six hours. Due to the interference of the colonoscopy, with no abnormal findings until and during its realization, we considered the intraoperative condition as a postcolonoscopy complication. The patient remained hemodynamically stable, still with normal temperature and blood laboratory results, and was discharged on the tenth postoperative day on good health condition.

## 3. Discussion

Diagnostic colonoscopy is considered a relatively safe procedure, with a limited range of serious complications. Acute appendicitis following colonoscopy is a rare complication, with a considered incidence of 0.038% [7], according to a research of 2007, either because of underpublication or underrecognition due to simultaneous conservative treatment.

For the review of the literature, we searched only the cases where an uncomplicated classic colonoscopy was performed, without signs of inflammation at the anatomic area of the appendix during the examination. There were no limitations of language or date of publication. An electronic literature search was performed on MEDLINE and manual retrieval of the reference lists of selected papers completed the research. A total of 31 articles were identified in the literature, with a total of 37 cases of postcolonoscopy appendicitis [5, 7–36]. The basic characteristics of these cases, including our own case, are included in Table 1.

The sample included 38 patients, 26 males (68.42%) and 12 females (31.58%). Mean age was 54.7 years (55.8 years for males, 52.3 years for females), with a range of 24 to 84 years. The time between the intervention and the presence of symptoms ranged from immediate to ten days after the procedure, with 26 patients (68.42%) showing symptoms within the first 24 hours after colonoscopy. Four patients (10.52%) were treated only conservatively; fifteen (39.47%) were submitted to exploratory laparotomy and appendectomy, seven (18.42%) to open appendectomy through McBurney's incision, and seven (18.42%) to laparoscopic appendectomy, whereas in five cases the exact treatment was not specified. Intraoperatively, acute appendicitis was diagnosed in ten cases, phlegmonous appendicitis in one case, gangrenous appendicitis also in one case, and perforated appendicitis in fourteen (36.84%), whereas nonspecified inflammation included six cases.

Over 68% of the cases have presented symptoms in a 24-hour period after the procedure, which suggests that there possibly exists an etiologic relationship. Four theoretical mechanisms have been proposed for the development of acute appendicitis after a colonoscopy: preexisting subclinical appendicitis, overinsufflation, impaction of feces or fecalith in the appendix, or direct trauma [15].

In our case, colonoscopy findings from the appendix were normal, so early appendicitis was most probably excluded from the differential diagnosis. Actually, colonoscopy is considered useful even in the diagnosis of appendicitis in cases where imaging studies are not diagnostic [37], so any possible inflammation of the appendix would have been directly shown during this examination.

We support that the causal event was the fecalith that could be introduced into the appendix by the air insufflation. This is suggested by no symptoms prior to the procedure, followed by the finding of impacted stool in the appendix after the open appendectomy. However, the possibility of the presence of the fecalith in the appendix prior to the colonoscopy could not be excluded, as we can also not exclude the fact that perforation could be due to overinsufflation or trauma from the scope. The presence of peritonitis could be explained by the interval of 20 hours from the moment of the presence of mild postcolonoscopy symptoms until the surgical management.

It could be suggested that the presence of fourteen cases of perforation intraoperatively could be due to a delay of diagnosis and deteriorated condition; however, even the immediate diagnosis and treatment [5] show that delayed diagnosis is not always the reason of complicated appendicitis. Even though delayed diagnosis was indeed a characteristic of many cases, immediate surgical procedures after the diagnosis of acute appendicitis could reduce the presence of intraoperative perforation, peritonitis, or any further deterioration of the inflammation.

CT is the examination of choice in order to obtain the optimal preoperative abdominal image, as postcolonoscopy abdominal pain can be generally caused by retained gas, colonic spasm, or postpolypectomy syndrome [7]. Exploratory laparotomy was the main form of operation in cases of diffuse inflammation; however, laparoscopic procedures have relatively recently been introduced and used in cases of perforated appendicitis [23, 27, 31] with good postoperative results.

## 4. Conclusion 

Postcolonoscopy appendicitis may be incidental or the result of the procedure. The mechanism of this complication is still unknown and theoretical. However, acute appendicitis should be always included in the differential diagnosis of postcolonoscopy abdominal pain, in order to avoid a delayed or complicated diagnosis and treatment.

## Figures and Tables

**Table 1:** Presentation of main characteristics of cases described in the literature (ns: not specified).

First author	Year	Sex	Age	Time after colonoscopy	Treatment	Condition
Houghton	1988	Male	35	8 hours	Laparotomy-appendectomy	Acute
Brandt	1989	Female	65	48 hours	Laparotomy-appendectomy	ns
Segawa	1992	Male	49	4 hours	ns	Perforated
Vender	1995	Female	44	Several hours	Laparotomy-appendectomy	ns
Vender	1995	Female	57	Immediate	Laparotomy-appendectomy	Perforated
Vender	1995	Male	55	48 hours	Antibiotics	ns
Hirata	1996	Male	69	12 hours	Laparotomy-appendectomy	Acute
Lipton	1999	Male	69	96 hours	Laparotomy-appendectomy	Gangrenous
de Leusse	1999	Male	71	12 hours	Laparotomy-appendectomy	Perforated
Takagi	2000	Male	56	72 hours	Laparotomy-appendectomy	Phlegmonous
Kapral	2003	Male	79	Same day	ns	Perforated
Srivastava	2004	Male	65	10 days	Laparotomy-appendectomy	Abscess
Rosen	2005	Male	24	48 hours	Laparotomy-appendectomy	ns
Rosen	2005	Male	55	Several hours	Antibiotics	ns
Izzedine	2005	Male	61	1 day	Antibiotics-appendectomy	Perforated
Volchok	2006	Male	60	16 hours	Appendectomy	Nonperforated
Chae	2007	Female	48	Same day	Appendectomy	Nonperforated
Horimatsu	2007	Male	68	1 day	Antibiotics	ns
Pellish	2007	Male	50	Immediate	Laparotomy-appendectomy	Nonperforated
Johnston	2008	Male	55	16 hours	Appendectomy	Perforated
Sheikh	2010	Female	50	4 hours	Laparotomy-appendectomy	Perforated
Moorman	2010	Female	71	Next morning	ns	Perforated
Moorman	2010	Male	47	27 hours	Laparotomy-appendectomy	Perforated
Moorman	2010	Male	84	4 hours	Laparotomy-appendectomy	Acute
Bachir	2010	Male	28	2 days	Appendectomy	Nonperforated
Penkov	2011	Female	50	7 days	Laparotomy-appendectomy	Acute
Rodriguez-Otero	2011	Female	50	6 hours	Laparotomy-appendectomy	Perforated
Aguilar-Shea	2011	Male	34	12 hours	ns	Acute
Louleiro	2011	Male	54	4 hours	Laparotomy-appendectomy	Perforated
Yagnik	2012	Female	35	7 days	Laparotomy-appendectomy	Acute
Musielac	2012	Female	45	4 hours	Laparotomy-appendectomy	Perforated
Musielac	2012	Male	33	6 hours	Laparotomy-appendectomy	Acute
Shaw	2013	Male	67	24 hours	Appendectomy	Acute
April	2013	Male	52	8 hours	Appendectomy	Nonperforated
Kuriyama	2014	Male	83	ns	ns	Acute
Wong	2014	Male	47	13 hours	Laparotomy-appendectomy	Perforated
Nemoto	2015	Female	53	7 hours	Antibiotics	Acute
Paramythiotis	2016	Female	60	24 hours	Laparotomy-appendectomy	Perforated
